# Can Cross-Sectional Imaging Reliably Determine Pathological Staging of Right-Sided Colon Cancers and Select Patients for More Radical Surgery or Neo-Adjuvant Treatment?

**DOI:** 10.7759/cureus.28827

**Published:** 2022-09-06

**Authors:** Florence Shekleton, Edward Courtney, Adrian Andreou, John Bunni

**Affiliations:** 1 Surgery, Royal United Hospital Bath, Bath, GBR; 2 General Surgery, Royal United Hospital Bath, Bath, GBR; 3 Radiology, Royal United Hospital Bath, Bath, GBR

**Keywords:** ct, colorectal cancer, staging, radiology, cme

## Abstract

Purpose and research question

Cross-sectional imaging with CT scanning is the most commonly performed imaging modality to stage right-sided colon cancers. There is increasing evidence for the use of neo-adjuvant chemotherapy in selected patients and debate about the role of complete mesocolic excision (CME) and central vascular ligation (CVL) in the management of locally advanced colon cancers. Predicted tumour stage and the presence of nodal metastases by CT are often used to select patients for neo-adjuvant chemotherapy and those that may benefit from CME.

This study aims to compare predicted radiological T and N staging with final pathological T and N staging in elective patients having potentially curative surgery for right-sided colon cancer.

Methods

A retrospective analysis was carried out of a prospectively gathered database of all patients who had undergone (true) right hemicolectomy between 02/01/13 and 21/05/20. Sensitivity, specificity, positive predictive value, and negative predictive value for CT scanning with regards to the pathological nodal metastases were calculated and analysed.

Results

The sensitivity and specificity of radiology staging for predicting nodal status were 76.4% and 65.5% respectively. The positive predictive value of CT staging for correctly identifying nodal metastases was 55.3%, with a negative predictive value of 77.3%.

Conclusions

This large series adds further evidence that CT, even when reviewed by expert GI radiologists, has limited accuracy at identifying lymph node metastases in colon cancer.

## Introduction

There are approximately 42,000 new cases of colorectal cancer diagnosed in the UK every year [1]. Despite the modern era of biological and immunological therapies and advances in radiotherapy, surgery remains the mainstay of treatment in the management of visceral cancers. Recent reports indicate that 80% of all cases of cancer will require operative intervention; in some cases, several times [2].

CT scanning is the most common method used to stage colon cancer within the UK and worldwide. A meta-analysis of 16 studies on the diagnostic accuracy of CT for staging nodal positivity of colon cancer showed a pooled sensitivity and specificity of 71% and 67% respectively [3]. In a more recent study focusing specifically on right-sided cancers, Fernandez et al. reported a sensitivity and specificity of 47% and 71% respectively for correctly predicting nodal disease [4]. Overall, the accuracy of CT for identifying any high-risk feature in right-sided colon cancers (pT3/T4, pN+, or EMVI+) was 62%. The authors concluded that CT may not be sufficient to identify patients pre-operatively who would benefit from either neo-adjuvant chemotherapy or more extensive nodal resection.

There is no doubt as to the primacy of mesenteric-based resectional surgery in the management of colon and rectal cancer [5]. With regards to rectal cancer, the use of total mesorectal excision (TME) and MRI for local staging has significantly reduced local recurrence, by accurately predicting patients with high-risk diseases and involved surgical margins. Increasingly, there is a focus on the benefits of selective complete mesocolic excision (CME) for high-risk colon cancers. Introduced by Hohenberger in 2009 [6], CME resonates with TME as it emphasizes strictly anatomical dissection along embryological planes to detach a perfectly intact mesentery with a peritoneal envelope housing the local field of cancer spread in an ontogenetic package. This also incorporates central vascular ligation (CVL) which broadly correlates with D3 lymphadenectomy. Hohenberger demonstrated that adoption of CME was associated with a reduction in rates of local recurrence (LR) rate from 6.5 to 3.6% and increased five-year cancer-related survival from 82.1% to 89.1% from 1978-1984 to 1995-2002 [6]. As it stands, no absolute indications for CME exist. The 2014 Japanese Society for Cancer of the Colon and Rectum (JSCCR) Guidelines for the treatment of colorectal cancer propose that any lymph node metastasis recognised before or during the surgery should undergo D3 lymph node dissection [7].

What is clear, is the importance of understanding the accuracy of CT in terms of correctly identifying involved nodes when planning treatment for patients with right-sided colon cancers in the multidisciplinary team. Herein, we examine our data with a primary outcome of assessing the validity of CT to predict final histology. We then discuss the use of CT staging in patient stratification for CME.

## Materials and methods

This was a retrospective analysis of a prospectively gathered database approved by the hospitals’ local audit and service provision department (ID 3576).

The database was used to identify all patients who had undergone right hemicolectomy between 02/01/13 and 21/05/20. These are defined as resections up to and including the right branch of the middle colic artery. Extended right hemicolectomy (defined as vascular ligation of the middle colic artery at its origin) and patients having non-curative surgery were excluded.

A total of 390 patients were identified from the database. Seven were excluded as they did not have a record of a pre-operative CT scan. Also, 269 patients had TNM staging in the original CT report which had been reviewed by a dedicated consultant gastrointestinal radiologist and had the radiological staging documented at the multi-disciplinary team (MDT) meeting. Of these 13 only contained T staging and 80 had staging which gave two values e.g. T2/3, and 114 had no staging in their pre-operative CT report or MDT.

All those without previous staging or staging giving two values were reviewed by a GI radiologist (AA) with nine years of consultant experience and a specialist interest in oncological and gastrointestinal imaging. These images were then staged or committed to one specific integer. The radiologist was blinded to the final histological staging. All CT studies were analysed using axial, coronal and sagittal sequences available at 1.5 mm slice thickness, with the ability to also do multiplanar reformats. 

**Figure 1:**
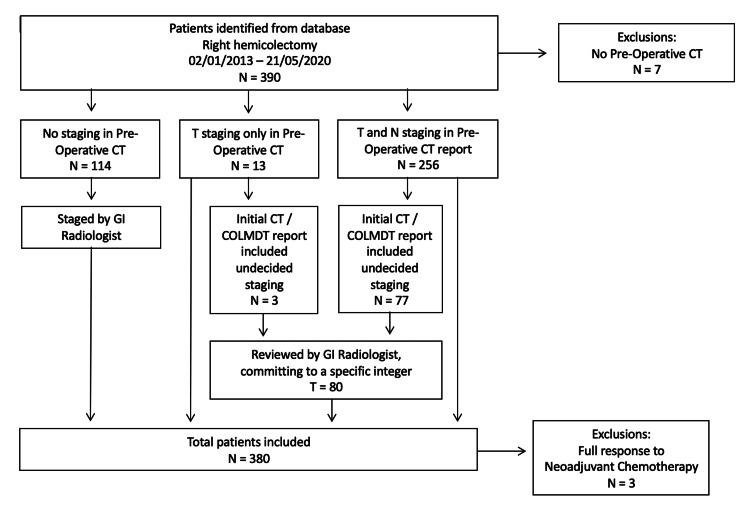
Methods flow chart (CT: Computerised Tomography, COLMDT: Colorectal Multidisciplinary Team Meeting)

A further three patients were then excluded as they had a complete response to neo-adjuvant chemotherapy and had a histological T stage of 0. TNM 7 was used. A total of 380 patients were used for the final data analysis. The full study strategy is shown in Figure 1.

Pre-treatment radiological stage was then cross-referenced with the final histopathology stage. SPSS Statistics (IBM Corp., Armonk, NY) was used for all data analysis and statistics.

## Results

Of the 380 patients included in the final analysis, 49.7% were male and 50.3% female. Around 88.1% of patients were between 60 and 89 years and 75.2% had a BMI between 20 and 29. Only 3.9% had neo-adjuvant chemotherapy as part of the FOxTROT trial ongoing during the study period and 36.6% had adjuvant chemotherapy. Additionally, 77% of the operations were elective (Table 1).

**Table 1:** Patient demographic data (n=380)

Demographic	Value	Frequency	%
Gender	Male	189	49.7%
	Female	191	50.3%
Age	20-29	1	0.3%
	30-39	7	1.8%
	40-49	4	1.1%
	50-59	26	6.8%
	60-69	62	16.3%
	70-79	154	40.5%
	80-89	119	31.3%
	90-100	7	1.8%
BMI	15-19	13	3.4%
	20-24	138	36.3%
	25-29	148	38.9%
	30-34	72	18.9%
	35-39	19	5.0%
	40-44	6	1.6%
	45-49	3	0.8%
Neoadjuvant chemotherapy	Yes	15	3.9%
	No	363	95.5%
	No data	2	0.5%
Adjuvant chemotherapy	Yes	139	36.6%
	No	204	53.7%
	No data	37	9.7%
Emergency	Yes	88	23%
	No	292	77%

The distribution of cancers by T stage is shown in Table 2, with the majority of resected cancers being T3 tumours (51.3%). Analysis of nodal staging showed that 58.4% of cancers had no evidence of lymph nodes metastases (Table 3).

**Table 2:** T stage distribution of cancers by pathological stage (n=380)

T stage	Frequency	Percentage
1	15	3.9
2	39	10.3
3	195	51.3
4	131	34.5

**Table 3:** Nodal status of patients (n=380)

N stage	Frequency	Percentage
0	223	58.4
1	92	24.2
2	66	17.4

**Figure 2:**
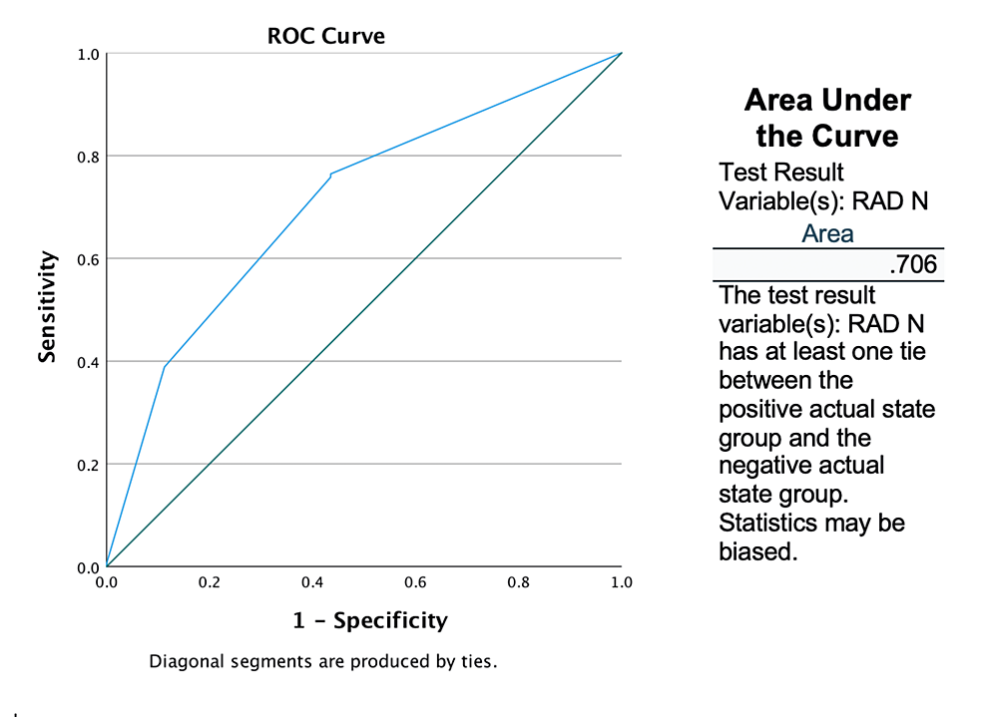
ROC curve and AUC ROC: receiver operating characteristic; AUC: curve area under the ROC curve

The sensitivity and specificity of radiology staging for predicating nodal status were 76.4% and 65.5% respectively (Table 4). The receiver operating characteristic (ROC) curve was used to determine the area under the ROC curve (AUC) of 0.706 (Figure 2). The positive predictive value of CT staging for correctly identifying nodal metastases was 55.3%, with a negative predictive value of 77.3%. The sensitivity and specificity for nodal status were only marginally altered when analysing according to BMI (BMI>30 vs BMI<30).

**Table 4:** Sensitivity and specificity of nodal staging by CT scan (n=380) Sensitivity = 120/(120+37)=76.4%, Specificity = 126/(126+97)=65.5% Positive Predictive Value = 120/(120+97) = 55.3%, Negative Predictive Value = 126/(126+37) = 77.3%

	Pathological nodes positive	Pathological nodes negative	Total
Radiological nodes positive	120	97	217
Radiological nodes negative	37	126	163
Total	157	223	380

The accuracy of radiological prediction of T1 tumours on final pathology was 53.3%, T2 tumours 56.4%, T3 tumours 59.5% and T3 tumours 49.6% (Table 5).

**Table 5:** Accuracy of radiology for predicting pathological T stage (n=380)

			Pathological T Stage				
			pT1	pT2	pT3	pT4	Total
Radiological predicted T stage	Rad1	n %	8 53.3%	6 15.4%	5 2.6%	2 1.5%	21 5.5%
	Rad2	n %	7 46.7%	22 56.4%	54 27.7%	10 7.6%	93 24.5%
	Rad3	N %	0 0%	10 25.6%	116 59.5%	54 41.2%	180 47.4%
	Rad4	N %	0 0%	1 2.6%	20 10.3%	65 49.6%	86 22.6%
Total			15	39	195	131	380

## Discussion

Our data shows that the positive predictive value of CT for identifying involved lymph nodes was 55.3%, meaning that CT was effectively not much better than "a coin toss" to differentiate node positive from node-negative disease. Given that all UK MDTs currently stage colon cancer using CT scanning, the importance of highlighting its limitations is paramount.

Limitations

It should be noted, that although an experienced gastrointestinal radiologist was used to retrospectively analyse previous imaging, it may have been beneficial to have a second radiologist re-stage all the CT scans and then assess the inter-rater reliability with Cohen’s kappa coefficient. However, given that 269 of the scans were already reviewed prior to this study, the majority of scans had been doubly reviewed in our study. Anyhow, it seems that analysis and comparison by more experienced radiologists may not have improved the lymph node staging from CT. Hong et al. have shown that although differentiation between T2 and T3 improves with experience, evaluation of lymph node involvement does not [8]. A study from the Karolinska institute [9] compared CT and pathology in 94 patients with colon cancers, reviewed by two blinded GI radiologists with >20 years of experience. For nodal metastasis, inter-observer agreement (Cohens Kappa) was 0.72. However, sensitivity for both in detecting lymph node metastasis was 69%. The main discrepancy was the under-staging of lymph node metastasis. The authors here also highlight histopathological examinations have shown nearly 50% of lymph nodes in colon cancer are below 5 mm. Large nodes may simply be reactive to inflammation rather than involved by a tumour. This again shows that whilst there may be a good correlation between radiologists, it is the limitation of CT at identifying involved nodes that limits the sensitivity of CT.

However, there appears to be no superior alternative imaging modality at present. A Korean study showed the sensitivity and specificity of FDG-PET/CT be inferior to CT for detecting metastases in regional lymph nodes in a cohort of 433 patients undergoing both CT and FDG-PET/CT prior to surgery for colorectal cancer [10]. Nerad et al. [11] evaluated the accuracy of MRI for local staging of colon cancer in 55 patients, with all scans evaluated blindly by two experienced abdominal MRI radiologists. The sensitivity and specificity for detecting nodal involvement (N0 versus N+) were 47% and 86% and 68% and 64% for each radiologist. Despite lymph nodes being clearly visible on diffusion-weighted images, this did not necessarily represent metastatic involvement, with benign nodes also showing high cellularity with high diffuse-weighted images as well.

The FOxTROT randomised trial of pre-operative chemotherapy prior to surgical resection of more locally advanced colon cancers focused on the T stage, with the poor prognosis group comprising CT radiological evidence of T4 or T3 disease with ³5mm extramural extension [12]. Pre-operative lymph node staging was not a selection criterion, on the basis of both data from the QUASAR1 study [13], supported by meta-analyses of other trials, indicating the proportional risk reduction in disease recurrence was similar in both node-positive and negative diseases. In fact, of the 354 patients in the control group in the FOxTROT trial who went straight to surgery, 48% were node-negative on their final histology. Conversely, only 6% had T2 disease, highlighting that CT is much better at correctly identifying patients with T3 and T4 diseases.

Kotake et al. [14] compared overall survival in a cohort of 6850 Japanese patients with T3 and T4 colon cancers. Patients undergoing a D3 resection had an 18% relative reduction in the risk of death compared to those having a D2 resection. Furthermore, subset analysis showed a greater chance of survival in patients having a D3 compared to D2 resection in node-negative colon cancer. This adds support to the hypothesis that removing the entire mesocolon removes micro-metastases present within the mesentery itself, not confined to lymph nodes or vessels.

Chen and Bilchik [15] showed that in patients with node-positive (Dukes C, stage III) colon cancer, their five-year overall survival (OS) increased from 67 to 90% when either 1-10 lymph nodes or more than 40 nodes were removed, respectively in N1 disease. They also showed an improvement in the five-year OS from 51-71% in N2 disease when either less than 35 lymph nodes or greater than 35 nodes were resected, respectively. Hohenberger et al. also reported that in node-negative patients, the five-year survival was significantly greater if 28 or more lymph nodes were removed [6].

Therefore, which group of patients should be offered a CME for right-sided colon cancers? Whilst nodal status is a hard indicator for CME, at least according to the Japanese guidelines, radiology is not a reliable discriminator for node-positive disease. The lack of radiological accuracy and consensus in staging colorectal cancers shown in this study and others means there are insufficient patient stratification methods. More accurate identification pre-operatively of T and N staging would allow selected patients to have neo-adjuvant chemotherapy and more radical surgery to hopefully improve outcomes.

## Conclusions

This large series adds further evidence to more limited published series of papers showing that CT, even when reviewed by expert GI radiologists, has limited accuracy at identifying lymph node metastases in colon cancer. More accurate imaging modalities are required to accurately stage tumour factors prior to surgery.

## References

[1] (2022). Cancer Research UK: Bowel Cancer Statistics. https://www.cancerresearchuk.org/health-professional/cancer-statistics/statistics-by-cancer-type/bowel-cancer.

[2] Sullivan R, Alatise OI, Anderson BO (2015). Global cancer surgery: delivering safe, affordable, and timely cancer surgery. Lancet Oncol.

[3] Nerad E, Lahaye MJ, Maas M, Nelemans P, Bakers FC, Beets GL, Beets-Tan RG (2016). Diagnostic accuracy of ct for local staging of colon cancer: a systematic review and meta-analysis. AJR Am J Roentgenol.

[4] Fernandez LM, Parlade AJ, Wasser EJ (2019). How reliable Is CT scan in staging right colon cancer?. Dis Colon Rectum.

[5] Bunni J, Coffey JC, Kalady MF (2020). Resectional surgery for malignant disease of abdominal digestive organs is not surgery of the organ itself, but also that of the mesenteric organ. Tech Coloproctol.

[6] Hohenberger W, Weber K, Matzel K, Papadopoulos T, Merkel S (2009). Standardized surgery for colonic cancer: complete mesocolic excision and central ligation--technical notes and outcome. Colorectal Dis.

[7] Watanabe T, Itabashi M, Shimada Y (2015). Japanese Society for Cancer of the Colon and Rectum (JSCCR) Guidelines 2014 for treatment of colorectal cancer. Int J Clin Oncol.

[8] Hong EK, Castagnoli F, Gennaro N (2021). Locoregional CT staging of colon cancer: does a learning curve exist?. Abdom Radiol (NY).

[9] Rollvén E, Blomqvist L, Öistämö E, Hjern F, Csanaky G, Abraham-Nordling M (2019). Morphological predictors for lymph node metastases on computed tomography in colon cancer. Abdom Radiol (NY).

[10] Yi HJ, Hong KS, Moon N, Chung SS, Lee RA, Kim KH (2014). Reliability of (18)f-fluorodeoxyglucose positron emission tomography/computed tomography in the nodal staging of colorectal cancer patients. Ann Coloproctol.

[11] Nerad E, Lambregts DM, Kersten EL (2017). MRI for local staging of colon cancer: can MRI become the optimal staging modality for patients with colon cancer?. Dis Colon Rectum.

[12] Seymour MT, Morton D (2019). FOxTROT: an international randomised controlled trial in 1052 patients (pts) evaluating neoadjuvant chemotherapy (NAC) for colon cancer. J Cancer Clin Oncol.

[13] Gray R, Barnwell J, McConkey C, Hills RK, Williams NS, Kerr DJ (2007). Adjuvant chemotherapy versus observation in patients with colorectal cancer: a randomised study. Lancet.

[14] Kotake K, Mizuguchi T, Moritani K, Wada O, Ozawa H, Oki I, Sugihara K (2014). Impact of D3 lymph node dissection on survival for patients with T3 and T4 colon cancer. Int J Colorectal Dis.

[15] Chen SL, Bilchik AJ (2006). More extensive nodal dissection improves survival for stages I to III of colon cancer: a population-based study. Ann Surg.

